# A Multicenter Phase II Trial of Docetaxel, Cisplatin, and Cetuximab (TPEx) Followed by Cetuximab and Concurrent Radiotherapy for Patients With Local Advanced Squamous Cell Carcinoma of the Head and Neck (CSPOR HN01: ECRIPS Study)

**DOI:** 10.3389/fonc.2019.00006

**Published:** 2019-01-22

**Authors:** Sadamoto Zenda, Yosuke Ota, Naomi Kiyota, Susumu Okano, Masato Fujii, Morimasa Kitamura, Shunji Takahashi, Tsutomu Ueda, Nobuya Monden, Takeharu Yamanaka, Makoto Tahara

**Affiliations:** ^1^Department of Radiation Oncology, National Cancer Center Hospital East, Kashiwa, Japan; ^2^Department of Radiation Oncology, Hyogo Cancer Center Hospital, Hyogo, Japan; ^3^Department of Medical Oncology, Cancer Center, Kobe University Hospital, Kobe, Japan; ^4^Department of Head and Neck Medical Oncology, National Cancer Center Hospital East, Kashiwa, Japan; ^5^Department of Otolaryngology, Tokyo Medical Center, Tokyo, Japan; ^6^Department of Otolaryngology, Head and Neck Surgery, Kyoto University Hospital, Kyoto, Japan; ^7^Department of Medical Oncology, Cancer Institute Hospital of JFCR, Tokyo, Japan; ^8^Department of Otorhinolaryngology-Head and Neck Surgery, Hiroshima University Hospital, Hiroshima, Japan; ^9^Department of Head and Neck Surgery, National Hospital Organization Shikoku Cancer Center, Matsuyama, Japan; ^10^Department of Biostatistics and Epidemiology, Yokohama City University School of Medicine, Yokohama, Japan

**Keywords:** head and neck cancer, induction chemotherapy, cetuximab, clinical trial, endpoint

## Abstract

**Background:** Induction chemotherapy (IC) is a treatment option for locally advanced squamous cell carcinoma of the head and neck (LA SCCHN). However, treatment with docetaxel, cisplatin, and 5-FU (TPF) followed by cisplatin and radiotherapy is controversial because of toxicity concerns. The aim of this phase II study was to assess the feasibility of docetaxel, cisplatin, and cetuximab (TPEx) followed by cetuximab and concurrent radiotherapy for LA SCCHN.

**Patients and Methods:** We enrolled patients with histological evidence of squamous cell carcinoma of the oropharynx, hypopharynx, or larynx without distant metastases. IC comprised cisplatin (75 mg/m^2^) and docetaxel (75 mg/m^2^) on day 1, repeated every 3 weeks for up to three courses. Cetuximab was initiated at 400 mg/m^2^, followed by 250 mg/m^2^ doses weekly until the end of radiotherapy. Radiotherapy (70 Gy/35 fr/7 w) was initiated after the last docetaxel administration. The primary endpoint was the rate of treatment completion.

**Results:** We enrolled 54 patients (median age, 58 years) between August 2013 and October 2015. Our patients were 49 males and 5 females with hypopharyngeal (*n* = 28), oropharyngeal (*n* = 19), or laryngeal (*n* = 7) cancers, and 48 of them had stage IV disease. The overall response rate was 72.2% with a median follow-up of 36.1 months and a 3-year overall survival of 90.7%. The treatment completion rate was 76%; 50 patients (93%) received ≥2 courses of IC, and 41 (76%) completed radiotherapy. The frequencies of grade ≥3 febrile neutropenia or allergy/infusion reactions were 39% and 11%, respectively. There was one treatment-related death.

**Conclusions:** IC with TPEx followed by cetuximab with concurrent radiotherapy showed acceptable compliance for the treatment of LA SCCHN. However, high frequency of febrile neutropenia remains a challenge and further improvement in the management of TPEx is necessary.

**Trial Registration:** UMIN000009928

## Introduction

Induction chemotherapy (IC) is a treatment option for locally advanced squamous cell carcinoma of the head and neck (LA SCCHN) and allows for organ preservation. Induction cisplatin and fluorouracil (PF) has been effective for locally advanced head and neck cancers before definitive radiotherapy (1, 2). In the GORTEC 2000–2001 study (3), induction docetaxel, cisplatin, and 5-FU (TPF) was superior to induction PF regimen in terms of the overall response rate. Moreover, in the TAX323 (4) and TAX324 (5) trials, induction TPF improved survival compared with induction PF. A recent meta-analysis of chemotherapy for head and neck cancer suggested that IC may contribute to control of distant metastases (6).

A docetaxel, cisplatin, and cetuximab (TPEx) regimen was tested as a first-line treatment for recurrent/metastatic HNSCC in the GORTEC 2008-03 study and showed good efficacy and compliance (7), suggesting that the TPEx regimen might be useful as IC. The TREMPLIN study comparing the efficacy and safety of IC followed by cisplatin or cetuximab with radiotherapy for larynx preservation (LP) showed that the regimen of cetuximab with radiotherapy achieved higher compliance (even after IC) than the cisplatin regimen, suggesting that it is one of the best options for LP.

We conducted a prospective phase II study to examine the feasibility of docetaxel, cisplatin, and cetuximab (TPEx) followed by cetuximab with concurrent radiotherapy for patients with LA SCCHN.

## Patients and Methods

This study was a multicenter, single-arm, phase 2 trial. Twenty-two institutions in Japan participated in this study. The study protocol was approved by the National Cancer Center Hospital Institutional Review Board. Written informed consents were obtained from all patients before enrollment in our study. This trial was registered with the UMIN clinical trials registry (UMIN000001439).

### Patients

We enrolled 54 patients with stage III-IV resectable locally advanced head and neck cancer fulfilling the following criteria: (1) histologically confirmed squamous cell carcinoma of the oropharynx, hypopharynx, or larynx; (2) age between 20 and 75 years; (3) Eastern Cooperative Oncology Group performance status between 0 and 1; (4) normal organ function; and (5) hope for organ preservation.

### Pretreatment Evaluation

Our pretreatment clinical evaluation included upper gastrointestinal and pharyngeal endoscopy; head and neck magnetic resonance imaging; and cervical, thoracic, and abdominal computed tomography (CT) scanning. Radiologists, surgeons, and oncologists evaluated the radiological lesion staging. We used the seventh edition of the International Union Against Cancer TNM classification for tumor staging. We did not routinely use positron emission tomography (PET) because of logistics (routine use of PET CT for staging and response evaluation was not accepted by government-issued health insurance).

### Protocol Treatment

The IC comprised intravenous (IV) administration of docetaxel (75 mg/m^2^) on day 1 and cetuximab (400 mg/m^2^ IV on day 1 of cycle 1, and 250 mg/m^2^ IV weekly on subsequent administrations) on days 1, 8, and 15. Cisplatin (75 mg/m^2^, IV) was also given on day 1. Cycles were repeated every 21 days thrice, with prophylactic antibiotics on days 5 through 14. We did not administer granulocyte colony-stimulating factor (G-CSF) prophylactically until November 2014, and prescribed it only in cases with febrile neutropenia (150 g/m^2^ per day). After a protocol revision on December 2014, we used prophylactic G-CSF for patients considered to be at a high risk for febrile neutropenia (8).

**Figure 1:**
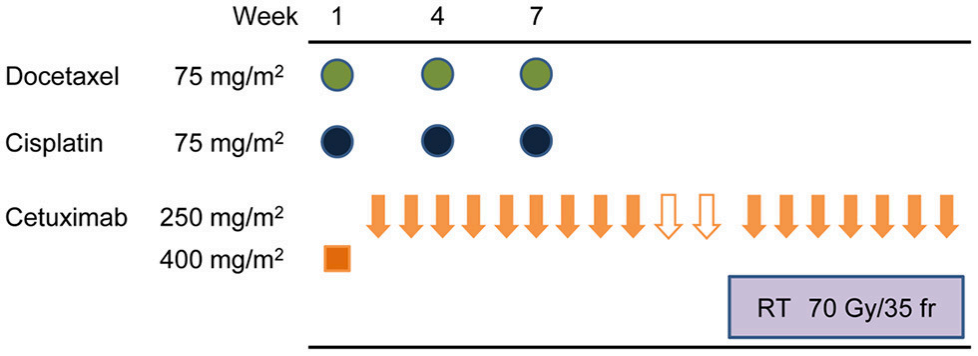
Protocol treatment schematic. Induction therapy comprised docetaxel (75 mg/m^2^) intravenously (IV) on day 1, cetuximab on days 1, 8, and 15, then cisplatin (75 mg/m^2^ IV) on day 1. Cycles were repeated every 21 days for three cycles. After three induction cycles, patients received standard radiotherapy, a total dose of 70 Gy in 35 fractions over 7 weeks with continued weekly cetuximab (250 mg/m^2^).

Two weeks after the second IC cycle, patients underwent endoscopies and CT scans of the neck and chest. Those with confirmed progressive disease (PD) stopped receiving the protocol treatment and received surgery or other appropriate treatments instead. Patients with confirmed non-PD status received a third IC cycle. After three IC cycles, all patients received standard radiotherapy (total dose of 70 Gy, in 35 fractions over 7 weeks with continued weekly cetuximab 250 mg/m^2^) (Figure 1).

Regarding the irradiation technique, both three-dimensional multi-beam irradiation (3D-RT) and intensity-modulated radiotherapy (IMRT) were accepted. We determined the gross tumor volume (GTV) by endoscopic or radiographic examination before the IC initiation. Clinical target volume (CTV) was defined as the GTV plus the volumes of all lesions considered at risk of containing microscopic disease. We further categorized the CTVs into two volumes, (1) a therapeutic CTV, including the primary tumor with a 1-cm margin craniocaudally and any metastatic nodes within a 0.5–2-cm margin, and (2) a prophylactic CTV, including a therapeutic CTV plus regional nodes. The planning target volume (PTV) was defined as the CTV plus a 1–3-mm margin that we adjusted as necessary when considering organ risk. The therapeutic and prophylactic PTVs received 70 and 40 Gy, respectively. We used five daily fractions of 2 Gy.

### Endpoints and Statistical Analyses

The primary endpoint was the treatment completion rate, which we identified in cases satisfying all of the following criteria: (1) patients received two or more IC courses; (2) irradiation was initiated within 6 weeks between the last IC course and the start of the radiotherapy; (3) full-dose irradiation was completed within 10 weeks; and (4) received cetuximab administration >12 times during their treatment.

In TAX 323 (4) and 324 (5) studies, the complete rates of induction chemotherapy in TPF group were 76 and 73%, respectively. Bonner et al. (9) reported the completion rate of cetuximab with radiotherapy was 90%.

Considering these and on the basis of 5% dropped out because of progressive disease between induction chemotherapy and radiotherapy, with regard to treatment completion rate as the primary endpoint in our phase 2 study, expected and threshold values for exact binomial test were 65 and 40%, respectively, and a total of 50 was required with a power of 90% and one-sided significance level of 2.5%.

We calculated the overall survival times from the date of study registration to the date of death, or the last confirmed survival date. We defined the progression-free survival (PFS) time from the study registration date until the first day of confirmation of PD at any site or of death by any cause.

Events for laryngo-esophageal dysfunction-free survival (LEDFS) included death, local relapse, total or partial laryngectomy or tracheotomy, and chronic enteral nutrition. We estimated binominal confidence intervals (CIs) for the overall response rate (ORR) by using the exact method and assessed the differences in these rates among subgroups using Fisher's exact test. We estimated survival curves using the Kaplan–Meier method.

We conducted primary analysis on the full analysis set population, defined as all registered patients excluding those ineligible after enrollment (i.e., those who did not receive any study treatment). We performed safety analysis for all registered patients who received at least one dose of study treatment. We performed all statistical analyses using the SAS software version 9.4.

## Results

### Patient Characteristics

**Figure 2:**
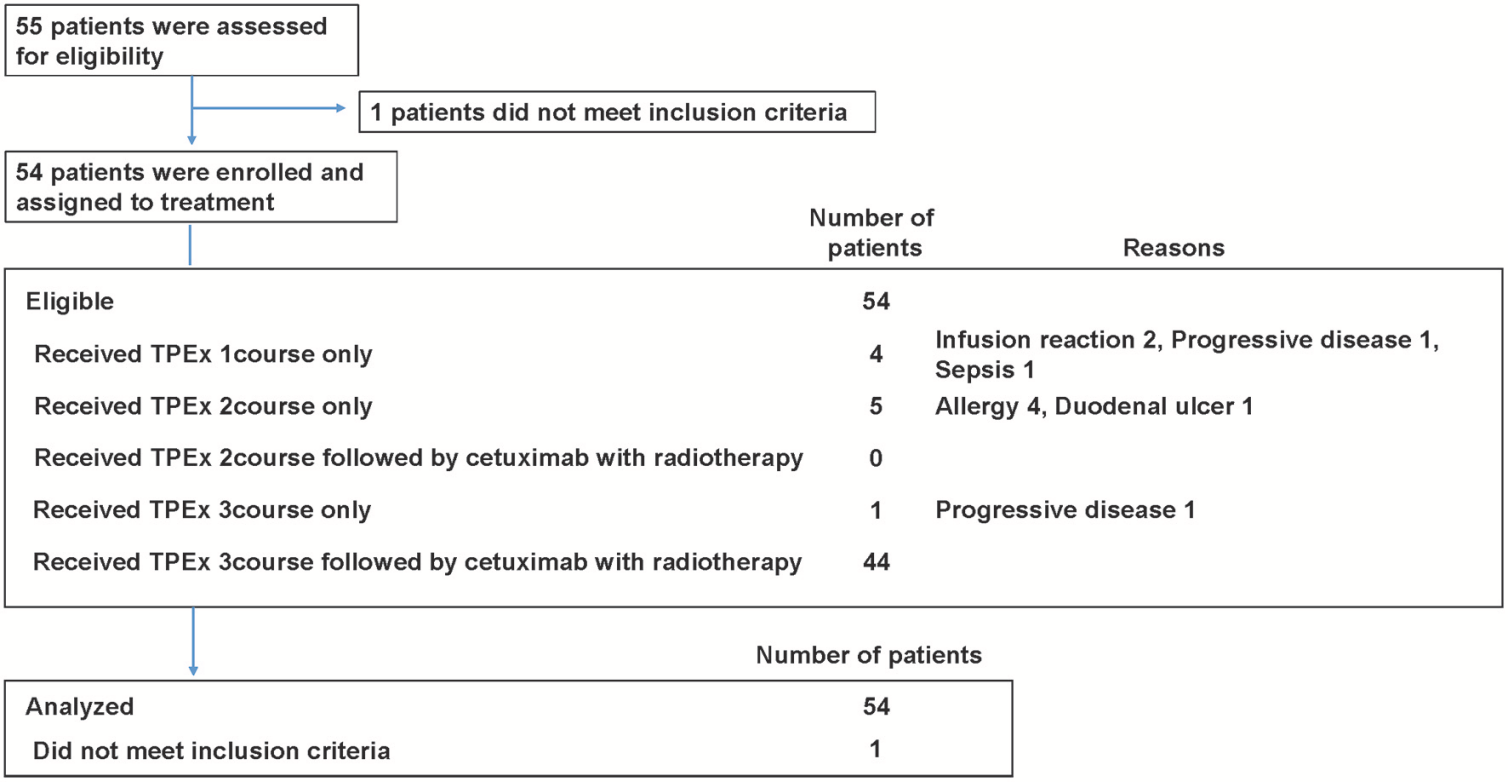
CONSORT diagram. Total 54 patients were analyzed in our study.

Patient characteristics are summarized in Table 1 and Table S1. Total 54 eligible patients with a median age of 58 years participated in the study (49 males and 5 females, 48 with stage IV disease) between August 2013 and October 2015 (Figure 2). The numbers of patients with hypopharyngeal, oropharyngeal, and laryngeal cancers were 28, 19, and 7, respectively. Of the 19 patients with oropharyngeal cancer, 14 had p16 positive oropharyngeal cancer.

**Table 1:** Patient characteristics (*n* = 54).

**Patient characteristics**
Age (years)		Median (range)	58 (35–72)
Sex		Male	49
		Female	5
Performance		0	42
Status		1	12
Primary site		Oropharynx	19
		Hypoparynx	28
		Larynx	7
TNM stage (7th edition)	T		
		1	1
		2	21
		3	12
		4	20
	N	0	8
		1	7
		2a	2
		2b	37
		3	0

### Treatment Compliance

The mean treatment completion rate was 75.9% (95% CI, 62.4–86.5%). The rate of patients receiving two or more TPEx cycles was 92.6% (50/54). Forty-four patients (44/54, 81.5%) received three TPEx cycles, and of those, 23 completed the planned TPEx.

The relative dose intensities of cisplatin and docetaxel were 0.90 (95% CI, 0.86–0.94) and 0.84 (95% CI, 0.80–0.88), respectively. The reasons for treatment interruption included seven severe adverse events (four allergies, two infusion reactions, and one sepsis), two PD cases, and other reasons (one duodenal ulcer). As a result, 10 patients could not receive radiotherapy with cetuximab within 6 weeks after the last course of TPEx.

Forty-four patients received radiotherapy, and of those, 41 patients (93.2%) completed the planned irradiation. The median radiotherapy duration was 51 days (range, 46–60). Reasons for treatment interruption included sepsis, local infection, and protocol deviation. Through IC and radiotherapy, the median times of cetuximab administration was 17 (range, 2–19), and the rate of patients receiving ≥12 administrations was 81.5% (44/54). Table 2 summarizes the treatment compliance results.

**Table 2:** Treatment compliance as the primary endpoint (*n* = 54).

	**Achievement rate in each category**
Induction chemotherapy (≥2 courses)	92.6%
Interval between the last administration of TPE and start of RT (< 6 weeks)	81.5%
Full-dose irradiation within 70 days	75.9%
Cetuximab administration (>12 times)	81.5%

*Total treatment compliance (all satisfied): 75.9% (95% CI: 62.4–86.5%)*.

### Toxicities

During the TPEx, the most frequent grade ≥3 toxicities were neutropenia (93%) and febrile neutropenia (39%). We modified our protocol during the study owing to the high frequency of grade ≥3 neutropenia and febrile neutropenia, and thus, initiated the administration of prophylactic G-CSF. The rate of febrile neutropenia dropped from 41.2 (14/34) to 35.0% (7/20) post protocol modification. Toxicity profile in TPEx is shown in Table 3. During radiotherapy, the most frequent grade ≥3 toxicities were mucositis (45%) and radiation dermatitis (48%). We did not find any infusion reactions or allergies during the radiotherapy phase, and we did not observe severe late toxicities during the follow-ups. Table 4 presents all grade toxicities during the radiotherapy phase.

**Table 3:** Toxicities at induction phase (*n* = 54).

	**Grade (CTCAE Ver 4.0)**, ***n***				**Grade 3–4, %**
	**1**	**2**	**3**	**4**	
**HEMATOTOXICITY**					
Neutropenia	0	2	15	35	93
Platelet	25	4	0	0	0
Anemia	36	11	4	1	9
**NON-HEMATOTOXICITY**					
Nausea	5	1	0	0	0
Anorexia	17	18	4	0	7
Mucositis	15	11	3	0	6
Skin rush	24	21	2	0	4
Infusion reaction	0	4	2	1	6
Allergy	0	2	4	1	9
Febrile neutropenia^*^	0	0	20	1	39

*CTCAE, Common Terminology Criteria for Adverse Events*.

* *ABx and G-CSF were allowed after protocol amendment, due to high rate of FN*.

**Table 4:** Toxicities at radiotherapy phase (*n* = 44).

	**Grade (CTCAE Ver 4.0)**, ***n***				**Grade 3–4, %**
	**1**	**2**	**3**	**4**	
**HEMATOTOXICITY**					
Neutropenia	7	2	0	0	0
Platelet	10	0	0	0	0
Anemia	28	11	3	0	7
**NON-HEMATOTOXICITY**					
Nausea	5	2	0	0	0
Anorexia	15	11	6	0	14
Mucositis	2	22	20	0	45
Skin rush	22	16	2	0	5
Infusion reaction	0	0	0	0	0
Allergy	0	0	0	0	0
Radiation dermatitis	2	18	21	0	48
Febrile neutropenia	0	0	0	0	0

*CTCAE, Common Terminology Criteria for Adverse Events*.

### Efficacies

**Figure 3:**
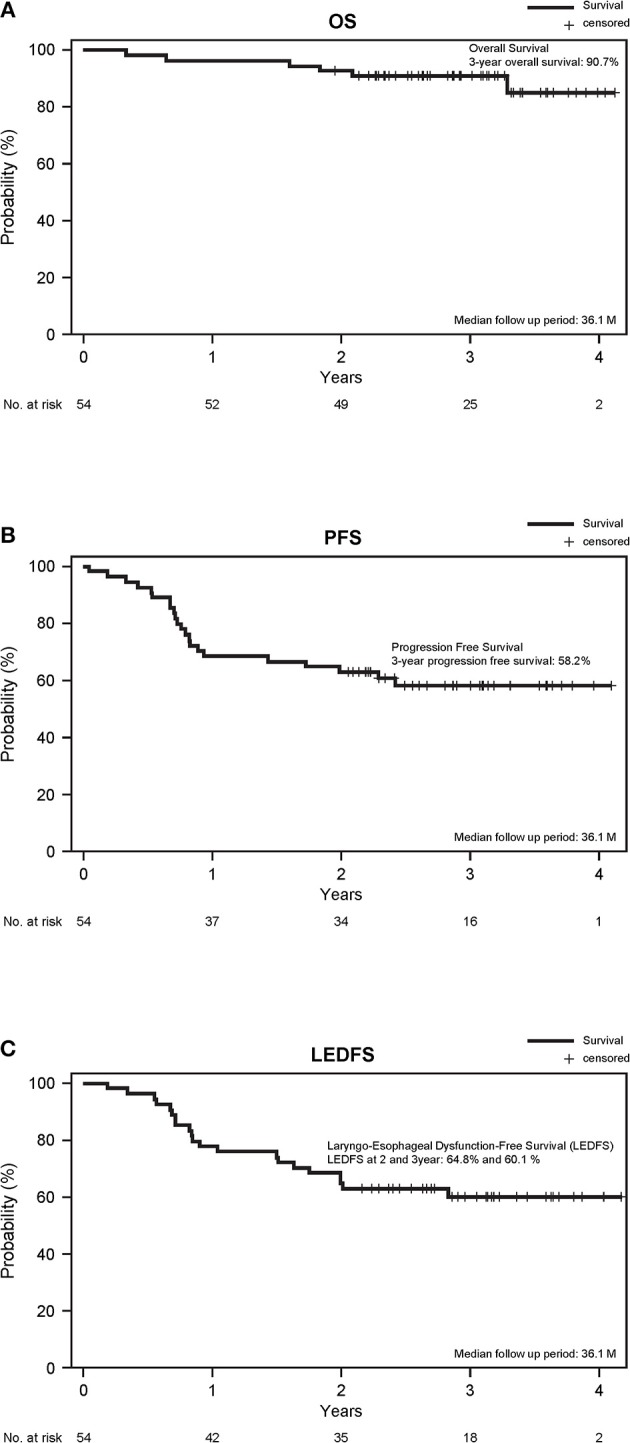
**(A)** Overall survival, **(B)** PFS, and **(C)** Laryngo-esophageal dysfunction-free survival. The survival time was calculated from the study registration date.

The ORR during the TPEx was 72.2% (95% CI, 58.4–83.5%). We observed complete responses (CR) in nine patients (16.7%), and one patient developed PD. The ORR after the radiotherapy was 75.9% (95% CI, 62.4–86.5%). We observed CR in 26 patients (48.1%), and 1 patient had PD. With a median follow-up period of 36.1 months, the 3-year overall survival and PFS rates were 90.7 and 58.2%, respectively. Twenty-six patients received second-line treatment: 11 patients underwent laryngectomy, 4 underwent neck dissection, 1 underwent surgery for lung metastases, 7 underwent chemoradiotherapy, 2 underwent chemotherapy, and 1 had incomplete data. The 2- and 3-year LEDFS were 64.8 and 60.1%, respectively (Figure 3).

## Discussion

TPEx followed by cetuximab with concurrent radiotherapy demonstrated an acceptable compliance for the treatment of LA SCCHN. However, high frequency of febrile neutropenia remains a challenge.

We used a cetuximab-based regimen instead of a platinum-based one. Induction TPF followed by chemoradiotherapy (TPF-CRT) seems to be the strongest regimen among the currently available regimens. However, patients have difficulty completing it, and thus, TPF-CRT is not the standard of care in the 2016 National Comprehensive Cancer Network guidelines due to concerns of toxicity and low compliance. Kim et al. (10) reported a meta-analysis of prospective trials (11–14), including TPF IC and chemoradiotherapy, stating that CRT treatment completion rate with this particular regimen was only 63.4% (478/651), and there was no statistically significant overall survival (OS) advantage for TPF prior to CRT (TPF/CRT) over CRT alone (hazard ratio [HR] 0.92; 95% confidence interval [CI], 0.79–1.09; *p* = 0.339).

Induction TPF with cetuximab (TPFE) was tested in the EORTC phase II study (15), showing a severe toxicity profile with only 63.8% (30/47) of patients reaching the radiotherapy phase. Therefore, the question of which IC is the best for the following chemoradiotherapy remains unanswered.

Considering the results of previous trials, we should consider the treatment compliance before discussing about efficacy.

Thus, the primary endpoint of this study was the treatment completion rate.

In this study, the rate of patients receiving two or more TPEx cycles reached 92.6%, and relative dose intensity of cisplatin and docetaxel were 0.90 (95% CI, 0.86–0.94) and 0.84 (95% CI, 0.80–0.88), respectively.

We observed a high frequency (39%) of febrile neutropenia in our patients, which appears to be one of the most important factors to be considered in a TPEx regimen. Because of this, we modified our protocol during the study and initiated the administration of primary and secondary prophylactic G-CSF. However, considering the small sample size, the frequency of febrile neutropenia appeared to be high even after protocol amendment. Thus, primary prophylactic G-CSF should be considered to manage the TPEx regimen.

Almost all of our patients completed planned irradiation. We encountered frequent cases of severe mucositis and dermatitis that we were able to control with standard oral care (16, 17) and nursing (18–20). We found no cases of infusion reaction or allergy during the radiotherapy phase and believe this may have been due to the gap of 2 months between the initial cetuximab administration and the radiotherapy initiation. The mean treatment completion rate was 75.9% (95% CI, 62.4–86.5%). As a result, seven patients of 10 patients who couldn't receive full dose radiotherapy had some trouble in TPEx section. Then, the management of TPEx section is of upmost importance.

A previous phase II study of TPEx conducted by Argiris et al. (21) showed similar results to ours and reported good compliance. A total of 39 patients were enrolled and of those, 35 patients (90%) received three cycles of cisplatin and docetaxel. A total of 34 patients (87%) received all planned doses of cetuximab during induction TPE, and 33 patients (85%) received full dose radiotherapy.

Several reports suggest that cetuximab with radiotherapy is not less toxic than chemoradiotherapy. However, the toxicity profile of cetuximab with radiotherapy was different from that of CDDP with radiotherapy and this point is important in considering the adjunctive treatment to IC.

The LP Consensus Panel recommended using LEDFS as a composite endpoint in preservation studies (22). In our phase II study, the 2-year LEDFS was 64.8%, greater than that reported in other studies (23, 24).

The 3-year OS and PFS rates were excellent at 90.7 and 58.2%, respectively. However, the efficacy of this regimen couldn't be discussed from these results, because 14 patients with p16 positive oropharyngeal cancer were also included.

Two recent phase III trials showed cetuximab with radiotherapy to be inferior to CDDP with radiotherapy in efficacy (25, 26); therefore, re-evaluation of TPEx followed by cetuximab with radiotherapy in efficacy is mandatory.

In conclusion, IC with TPEx followed by cetuximab with concurrent radiotherapy showed acceptable compliance for the treatment of LA SCCHN. However, high frequency of febrile neutropenia remains a challenge and further improvement in the management of TPEx is necessary.

## Author Contributions

SZ, NK, MF, TY, and MT: study concept. SZ, NK, TY, and MT: study design. SZ, YO, NK, SO, MF, MK, ST, TU, NM, and MT: acquisition of data. TY: analysis of data. SZ, NK, ST, and MT: interpretation of data. NK, ST, TY, and MT: drafting of the manuscript. SZ, YO, SO, MF, MK, TU, and NM: critical revision of the manuscript. All authors read and approved the final version of the manuscript.

### Conflict of Interest Statement

SZ reports personal fees from Merck Serono during the conduct of the study. MT reports personal fees from Merck Serono, grants and personal fees from MSD, Bayer, Eisai, Pfizer, Astra Zeneca, Ono Pharmaceutical and Bristol-Myers Squibb, personal fees from Otsuka, grants from Boehringer-Ingelheim, Novartis and NanoCarrier, outside the submitted work. NK reports honoraria from Merck Serono, grants from research funding from Ono Pharmaceutical, and Astra Zeneca, and honoraria from Ono Pharmaceutical, Bristol-Meyers Squibb, Astra Zeneca, Eisai and Bayer. Outside the submitted work, ST reports grants and personal fees from Takeda, grants and personal fees from Taiho, personal fees from Boehringer-Ingelheim, personal fees from Chugai. Outside the submitted work, ST reports honoraria from Merck Serono during the conduct of the study; grants and personal fees from Bristol-Meyers Squibb and MSD. The remaining authors declare that the research was conducted in the absence of any commercial or financial relationships that could be construed as a potential conflict of interest.

## Abbreviations

CI: Confidence intervals
CR: Complete responses
CSPOR: Comprehensive Support Project of the Public Health Research
CT: Computed tomography
CTV: Clinical target volume
GETTEC: Groupe d'Etude des Tumeurs de la Tete et du Cou
GTV: Gross tumor volume
IC: Induction chemotherapy
LEDFS: Laryngo-esophageal dysfunction-free survival
LP: Larynx preservation
ORR: Overall response rate
PD: Progressive disease
PET: Positron emission tomography
PFS: Progression-free survival
PTV: Planning target volume.
