# Supplementary material for: A Multicenter Phase II Trial of Docetaxel, Cisplatin, and Cetuximab (TPEx) Followed by Cetuximab and Concurrent Radiotherapy for Patients With Local Advanced Squamous Cell Carcinoma of the Head and Neck (CSPOR HN01: ECRIPS Study)
Source: Front Oncol. 2019 Jan 22;9:6. doi: 10.3389/fonc.2019.00006 (PMC6349830; doi:10.3389/fonc.2019.00006)
Supplement: Supplementary file 1 [file Table_1.DOCX]

Supplementary Appendix: List of Investigators

| Name | Institution |
| --- | --- |
| Sadamoto Zenda | Department of Radiation Oncology, National Cancer Center Hospital East, Kashiwa, Japan |
| Makoto Tahara | Department of Head and Neck Medical Oncology, National Cancer Center Hospital East, Kashiwa, Japan |
| Masato Fujii | Department of Otolaryngology, Tokyo Medical Center, Tokyo, Japan |
| Yosuke Ota | Department of Radiation Oncology, Hyogo Cancer Center, Hyogo, Japan |
| Susumu Okano | Department of Head and Neck Medical Oncology, National Cancer Center Hospital East, Kashiwa, Japan |
| Hirofumi Fujii | Department of Clinical Oncology, Jichi Medical University Hospital, Shimotsuke, Japan |
| Ichiro Ota | Department of Otolaryngology, Head and Neck surgery, Nara Medical University Hospital, Kashihara, Japan |
| Tomoya Yokota | Division of Gastrointestinal Oncology, Shizuoka Cancer Center, Shizuoka, Japan |
| Akihiro Homma | Department of Otolaryngology, Hokkaido University Hospital, Sapporo, Japan |
| Shunji Takahashi | Department of Medical Oncology, Cancer Institute Hospital of JFCR, Tokyo, Japan |
| Takashi Toshiyasu | Department of Radiation Oncology, Cancer Institute Hospital of JFCR, Tokyo, Japan |
| Nobuya Monden | Department of Head and Neck surgery, National Hospital Organization Shikoku Cancer Center, Matsuyama, Japan |
| Nobuhiro Hanai | Department of Head and Neck Surgery, Aichi Cancer Center, Nagoya, Japan |
| Naomi Kiyota | Department of Medical Oncology and Hematology, Kobe University Hospital Cancer Center, Kobe, Japan |
| Kazuhiko Nakagawa | Department of Medical Oncology, Kindai University Hospital, Osaka, Japan |
| Morimasa Kitamura | Department of Otolaryngology, Head and Neck surgery, Kyoto University Hospital, Kyoto, Japan |
| Kyoya Kumagaya | Division of Hematology-Oncology, Chiba Cancer Center, Chiba, Japan |
| Masaki Kokubo | Department of Radiation Oncology, Kobe City Medical Center General Hospital, Kobe, Japan |
| Tsutomu Ueda | Department of Otorhinolaryngology-Head and Neck Surgery, Hiroshima University Hospital, Hiroshima, Japan |
| Tetsuo Akimoto | Department of Radiation Oncology, National Cancer Center Hospital East, Kashiwa, Japan |
| Kenji Ookami | Department of Otolaryngology, Tokai University Hospital, Isehara, Japan |
| Takeshi Kodaira | Department of Radiation Oncology, Aichi Cancer Center, Nagoya, Japan |
